# Fluid therapy in patients with brain injury: what does physiology tell us?

**DOI:** 10.1186/cc13764

**Published:** 2014-03-12

**Authors:** Christian Ertmer, Hugo Van Aken

**Affiliations:** 1 Department of Anesthesiology, Intensive Care and Pain Therapy, University Hospital of Münster, Münster D-48149, Germany

## 

The unique component of the cerebral circulation is the so called blood–brain barrier (BBB). The anatomical structures of the BBB consist of the cerebral vascular endothelial cells, the surrounding pericytes, the basal lamina and the perivascular astrocytes. These form the so-called neurovascular unit [1]. Notably, the endothelial cells are interconnected by tight junctions; thus, any solute transport will be transcellular, as opposed to paracellular, in the peripheral circulation [2]. The specific anatomy of the neurovascular unit allows the brain volume to be kept constant even in the context of marked changes in intravascular volume status.

On the contrary, the BBB has a considerable passive permeability to free water, as opposed to electrolytes and other solutes. An acute drop in plasma osmolality, therefore, results in an acute increase in brain water content [3]. The neuronal cells compensate for the increase in volume by active depletion of intracellular osmotic solutes (so called ‘volume regulatory decrease’) [4,5]. Thus, excessive water moves to the extracellular space, thereby normalizing cellular volume. When plasma hypo-osmolality eventually resolves, brain water content proportionally decreases, which may lead to demyelinisation in severe cases [6]. Again, the cells react by internalizing osmotic solutes (‘volume regulatory increase’). However, this process is much less efficient than the depletion of solutes [7]. In summary, the critical sequelae of acute changes in osmolality imply that such alterations should be avoided whenever possible in the clinical setting.

The complex regulation of cerebral volume in response to changes in osmolality is of central interest in the context of fluid therapy in patients with intact or disrupted BBBs. The physical composition of resuscitation fluids is of special relevance in this regard [8]. Key determinants include the osmolality and the colloid osmotic pressure of the fluid preparation. In this context, it is of major importance to discriminate between the theoretical osmolarity, which reflects the sum of all potentially dissociable particles expressed in mosmol/l. Conversely, the osmolality represents the number of osmotically active solutes (in mosmol/kg) and thus is the clinically relevant variable. It may either be calculated from the theoretical osmolarity, the water content of the solution and the osmotic coefficient of the solute, or directly measured by freezing point depression. Notably, due to incomplete dissociation of soluble molecules, osmolality is lower than osmolarity in resuscitation fluids.

An iso-osmotic crystalloid fluid (that is, equalling the physiological plasma osmolality of 288 ± 5 mosmol/kg) equally distributes to the intravascular and the interstitial space [9], because the peripheral endothelial cells allow a more or less unrestricted exchange of water and electrolytes. Thus, large amounts of crystalloids are inevitably associated with a dose-dependent formation of extracellular oedema. Notably, the neurovascular unit prevents electrolytes from passively passing between the intravascular space and extravascular space [2]. Therefore, the intracranial volume is not increased even with large amounts of iso-osmotic crystalloid solutions. Hypo-osmotic solutions, in turn, distribute to the whole body water, including the intracellular space [10]. As discussed above, the brain will respond with an initial increase in cellular volume followed by active (thus ATP-consuming) depletion of intracellular solutes. Notably, patients with cerebral pathologies may not be able to compensate for the increase in cellular volume or the associated increase in cerebral oxygen consumption. To prevent the potentially lethal sequelae of brain oedema, hypo-osmotic solutions should generally be avoided for bolus or high-dose resuscitation or in patients with brain pathology.

In an ideal model of the vasculature, iso-oncotic colloid solutions remain in the intravascular space, since the endothelial barrier is not permeable to colloidal compounds [11,12]. In clinical reality, however, the volume effect of colloids is context-sensitive, depending on volume status and the presence of systemic inflammation [13,14]. Notably, infusion of colloids should not exert an intrinsic effect on the brain itself beyond its impact on the cerebral circulation. In this context, it is surprising that a clinical comparison of crystalloids (saline 0.9%) and colloids (4% human albumin), the Saline and Albumin Fluid Evaluation (SAFE) study, showed a less favourable outcome (higher 28-day mortality) for patients with traumatic brain injury treated with 4% human albumin [15,16]. This finding led to the recommendation that colloids should not be used in patients with head injury [17]. When having a closer look at the study drugs, isotonic saline has an osmolality of 286 mosmol/kg, and is thus iso-osmotic. The albumin preparation used in the SAFE study (Albumex 4%, CSL Ltd, Parkville, Victoria, Australia) however, has a nominal osmolality of only 260 mosmol/kg. Measurement of the freezing point depression revealed a measured osmolality of 266 mosmol/kg [8]. However, it is known that the freezing point depression method may overestimate the actual osmolality due to ‘cryoscopic colloid effects’. Taken together, the investigated colloid of the SAFE study represents a severely hypo-osmotic solution. When taking the above mentioned physiologic considerations into account, the SAFE study confirms that hypo-osmotic solutions are deleterious in patients with brain injury, rather than evaluating the colloid compound itself. Notably, these data have been misinterpreted several times in the current literature [16-18]. Given this, there is no reliable evidence that colloids themselves are hazardous in patients with brain injury. It is nevertheless important to consider the osmolality of each product prior to their use. Table 1 gives an overview of the physical properties of many resuscitation fluids.

**Table 1:** Physicochemical characteristics of available resuscitation fluid preparations

	**Colloid**	**Specific gravity (g/ml)**	**H**_**2**_**O content**	**Osmotic coefficient**	**Theoretical osmolarity (mosmol/l)**	**Real osmolality**^**a **^**(mosmol/kg)**	**Tonicity**
Plasma	Proteins	1.0258	0.940	0.926	291	287	Isotonic
NaCl 0.9%	None	1.0062	0.997	0.926	308	286	Isotonic
Dextrose 5%	None	1.0197	0.970	1.013	278	290	Isotonic (only *in vitro*)^b^
Ringer’s lactate	None		0.997	0.926	276	256	Hypotonic
Ringer’s acetate	None		0.997	0.926	276	256	Hypotonic
Plasmalyte®	None		0.997	0.926	294	273	Hypotonic
Sterofundin® ISO/Isofundin®/Ringerfundin®^d^	None		0.997	0.926	309	287^c^	Isotonic
Voluven® 6%	6% HES 130/0.4	1.0274	0.958	0.926	308	298	Hypertonic (slightly)
Volulyte® 6%	6% HES 130/0.4	1.0274	0.956	0.926	287	278	Hypotonic (slightly)
Venofundin® 6%	6% HES 130/0.42	1.0257	0.957	0.926	308	298	Hypertonic (slightly)
Tetraspan® 6%	6% HES 130/0.42	1.0257	0.955	0.926	296	292^b^	Isotonic
Gelafundin® 4%	4% polygeline	1.0177	0.969	0.926	274	262	Hypotonic
Gelafundin® ISO 4%	4% polygeline		0.969	0.926	284	271	Hypotonic
Albumex® 4%	4% human albumin		0.958	0.926	269	260	Hypotonic
Alburex® 5%	5% human albumin		0.948	0.926	281	274.5	Hypotonic (slightly)

^a^Osmolality reflects the calculated, nominal osmolality (as calculated from osmolarity, water content and osmotic coefficient). ^b^Since glucose is quickly metabolized and moved to the intracellular compartment, dextrose solutions behave severely hypotonic *in vivo*. ^c^Considering that one malate anion is metabolized to two hydrogen carbonate anions. ^d^Sterofundin ISO, Isofundin and Ringerfundin represent different labels for equivalent solutions. HES, hydroxyethyl starch. Modified from Physioklin [19].

In summary, changes in plasma osmolality may be deleterious in patients with brain injury. Resuscitation fluids should therefore be isotonic in terms of osmolality (not osmolarity). Physicians should be familiar with the osmolality of the resuscitation fluids they use.

### Abbreviations

BBB: Blood–brain barrier; SAFE: Saline and albumin fluid evaluation.

### Competing interests

The authors declare that they have no competing interests.
